# Acute inflammatory profile of patients submitted to pulmonary resection

**DOI:** 10.1590/acb401625

**Published:** 2025-02-03

**Authors:** Andrea Pelicia Roso de Souza, Raquel Palhares, Letícia Baltieri, Ricardo Kalaf Mussi, Edson Antunes, Glaucia Coelho de Mello

**Affiliations:** 1 Universidade Estadual de Campinas – Faculty of Medical Sciences – Campinas (SP) – Brazil.; 2 Universidade Estadual de Campinas – Faculty of Medical Sciences – Department of Surgery – Campinas (SP) – Brazil.; 3 Universidade Estadual de Campinas – Faculty of Medical Sciences – Department of Pharmacology – Campinas (SP) – Brazil.

**Keywords:** Inflammation, Thoracic Surgery, Interleukins, Cytokines

## Abstract

**Purpose:**

To establish a profile of the inflammatory response in the preoperative and postoperative period of pulmonary resection of patients without postoperative complications, in order to trace the inflammatory profile of lung resection surgery.

**Methods:**

Six collections of arterial and venous blood were performed for data analysis, one sample in the preoperative, immediate postoperative, 4, 8, 24, and 48 hours after surgery. Twenty-seven patients with a median age of 63 years old, ranging from 29 to 80 years old, were included.

**Results:**

The leukocyte count showed a significant increase in the times: immediate postoperative and 4 hours after surgery, in relation to the preoperative period. Concomitantly, there was an increase in lactate, heart rate, interleukin (IL)-6 and IL-8 after 4 hours of surgery. The platelet count showed a significant decrease in 48 h, associated with an increase in IL-1β and tumor necrosis factor-α. A significant increase in IL-10 was observed in the immediate postoperative.

**Conclusion:**

The study may contribute to the search for more specific and adequate alternatives for controlling the inflammatory response. In this way, the intervention would be specific to that cytokine that causes the greatest harm to the patient, as well as to the moment of the intervention.

## Introduction

Pulmonary resection is indicated for the treatment of patients with benign and malignant lung conditions in those who can support this therapy^1^. The incidence of acute lung injury in the postoperative period of pulmonary resection was related to be 2.8% with 33.3% of mortality^2^. Patients undergoing pulmonary resection develop an inflammatory process through numerous mechanisms including ventilation-induced lung injury, surgical trauma that are also involved in the development of acute lung injury^3^.

The inflammatory process is seen as a defense mechanism of the organism, but the inflammation can be potentially harmful, since its manifestation can harm the organism itself, in a more harmful way than the insulting agent^4^.

Systemic leukocyte activation (mediated by cytokines) is a direct manifestation of the generalized inflammatory response and, when excessive, can lead to multiple organ failure as a consequence of an exaggerated response to the systemic inflammatory response syndrome^5^.

However, there are still no reports in the literature on the interleukin (IL) release profile in the inflammatory response after pulmonary resection surgery without complications. Thus, the objective of this study was to establish a profile of the release of tumor necrosis factor-α (TNF-α), IL-1β, IL-6, IL-8, and IL-10. This study may allow a better understanding of the baseline inflammation profile in patients after pulmonary resection and consequently more effective interventions in perioperative period to modulate inflammatory response and its local or remote repercussion.

## Methods

The study was carried out in a prospective way and involved 27 patients admitted to the Hospital de Clínicas of Universidade Estadual de Campinas (UNICAMP), by the Thoracic Surgery Division submitted to pulmonary resection, varying in segmentectomy, lobectomy or pneumonectomy, during the period from 2012 to 2014.

The study was approved by the Ethics and Research Committee of the Faculty of Medical Sciences of UNICAMP, number 933/2009, and obtained financial resources granted by the São Paulo Research Foundation, process number: 2011/51873-9.

The population of patients included in this study was of both genders, aged 29 to 80 years old, hemodynamically stable, without the use of vasoactive drugs or pre- and postoperative metabolic and electrolytic disorders, without continuous or intermittent sedation after surgery and without postoperative complications.

Patients who developed postoperative hemodynamic instability, renal failure, pulmonary embolism, aspiration bronchopneumonia, and severe respiratory disorders were excluded from the research. Patients in the present study should have been without prior chemotherapy and radiotherapy for at least three months. There were no missing data in the present study.

The number of participants in the study was chosen based on the number of surgeries performed by the thoracic surgery team at the hospital per week during the study period, and the types of resections were performed according to the needs of each patient. Only those who presented the complications mentioned above were excluded. Surgery time ranged from 2 to 6,5 hours. The patients’ bodies were kept warm, and the fluids administered were also warmed throughout the surgery.

Data such as gender, race, body mass index, smoking, presence of associated diseases, use of corticosteroids or bronchodilators, type of neoplasia and stage, adjuvant treatment with chemotherapy or radiotherapy, presence of metastasis, existence of complications up to 48 hours after surgery, and need for antibiotic therapy were collected from the patient’s medical records.

In the pre- and postoperative period, the number of leukocytes and platelets, circulating levels of TNF-α, IL-1β, IL-6, IL-8, IL-10, arterial and venous blood gases, PaO_2_/FiO_2_, respiratory rate (RR), heart rate (HR), and body temperature were evaluated.

Six samples of arterial and venous blood were collected in the preoperative period, at the end of the surgery, 4, 8, 24 and 48 hours of postoperative, following the same time collection model as Yim et al.^6^ Arterial blood was used for blood gas analysis, and venous blood was divided into two tubes, one aliquot for the quantification of leukocytes and the other centrifuged and cooled to -80°C. The levels of TNF-α, IL-1β, IL-6, IL-8, IL-10 in plasma were analyzed using Multiplex analysis kits (microtests).

### Statistical analysis

Descriptive statistics were performed, with data presented in absolute and relative values and age in median and variation. For data analysis, the paired T test and analysis of variance associated with the Bonferroni’s test were applied, considering *p* < 0.05 as significant. The software used was Statistical Package for Social Sciences, version 14.0.

## Results

Twenty-seven patients were included in the study from December 2012 to April 2014 with a median age of 63 years old, 59% of whom were male. Among the data proposed for analysis, there was no missing data. Table 1 shows age, demographic characteristics, and types of lung resection and smoking history.

**Table 1:** Patient characteristics.

Variables	N = 27	%
Median age (range)	63 (29–80)	
Median body mass index (range)	23.21 (18.75–47.3)	
**Gender**		
Male	16	59.3
Female	11	40.7
**Type of pulmonar resection**		
Segmentectomy	6	22.2
Lobectomy	17	62.9
Bilobectomy	3	11.1
Pneumectomy	1	3.7
**Smoking history**		
Current smoker	7	25.9
Ex-smoker	7	25.9
Non-smoker	12	44.4
Passive smoker	1	3.7

Source: Elaborated by the authors.

**Figure 1:**
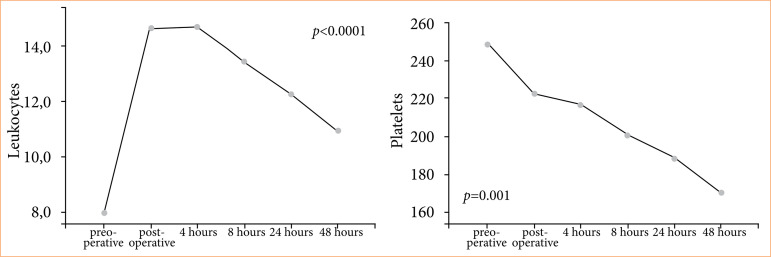
Leukocyte and platelet measurements over time.

Source: Elaborated by the authors.

Figure 1 describes the leukocyte and platelet measurements, with a significant increase in leukocytes (*p* < 0.0001) in the immediate postoperative period and 4 hours after surgery with gradual reduction afterwards, without returning to baseline. Platelets showed a significant reduction (*p* = 0.001) after 48 hours of surgery.

**Figure 2:**
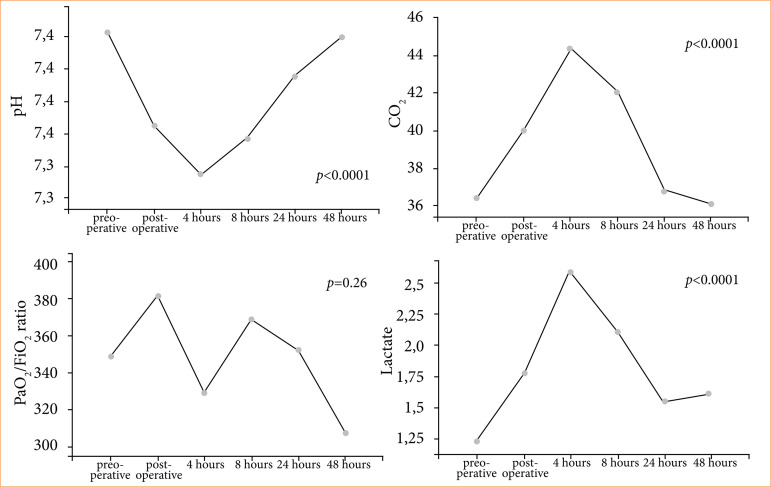
Measurements obtained from arterial blood gases, with pH, PCO2, PaO2/FiO2 ratio and lactate.

Source: Elaborated by the authors.

Figure 2 shows the data obtained from arterial blood gases, with a significant reduction in pH (*p* < 0.0001) and a significant increase in PCO_2_ (*p* < 0.0001) after 4 hours of surgery with recovery in 48 hours after. There was no significant difference in the PaO_2_/FiO_2_ ratio. Lactate showed a significant increase (*p* < 0.0001) after 4 hours of surgery.

**Figure 3:**
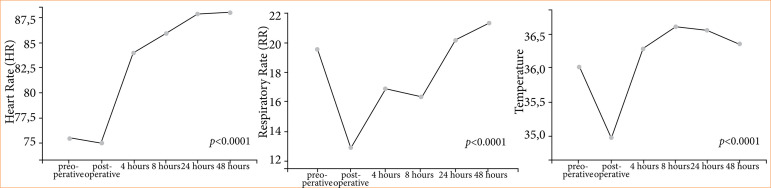
Heart rate, respiratory rate, and temperature over time.

Source: Elaborated by the authors.

Figure 3 shows a significant increase in heart rate (*p* < 0.0001) after 4 hours of surgery in relation to the preoperative baseline. There was also a significant decrease (*p* < 0.0001) in respiratory rate in the immediate postoperative period, in addition to a significant decrease (*p* < 0.0001) of temperature in the immediate postoperative period.

**Figure 4:**
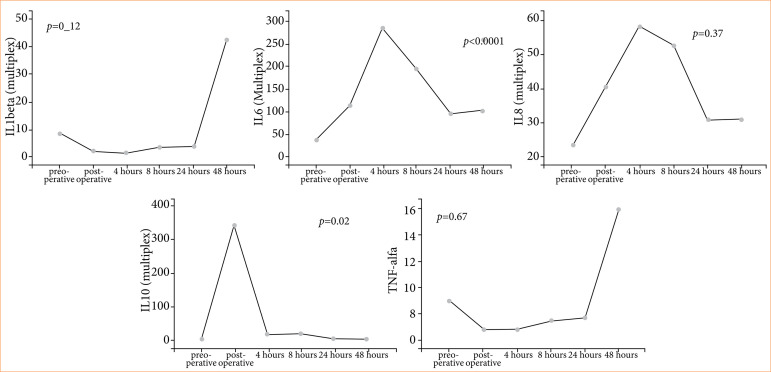
Interleukins (IL) over time.

Source: Elaborated by the authors.

Figure 4 shows the variation of interleukins and TNF-α. Although IL-1 beta showed a significant increase after 48 hours of surgery, there was no significant difference (*p* = 0.12). IL-6 showed a significant increase (*p* < 0.0001) between the preoperative period and after 4 hours of surgery with a significant decrease and returned to baseline levels after 24 hours (*p* = 0.07); IL-8 showed an increase after 4 hours of surgery, but without significant difference (*p* = 0.37); IL-10 showed a significant increase (*p* = 0.02) in the immediate postoperative period and TNF-α increased after 48 hours of surgery, but without significant difference (*p* = 0.67).

## Discussion

Background postoperative pulmonary complications have a significant clinical and economic impact associated with the increased observed number of deaths, morbidity, length of stay, and associated cost^7^. Evidence indicates that proinflammatory cytokines caused by surgical trauma play an important role in the development of postoperative complications^6^.

Cytokine expression and release in the circulation are common events in systemic inflammatory states. The standard proinflammatory cytokines, such as TNF-α, IL-1β, and IL-6, have been used as indicators of systemic inflammation^8^. Among these cytokines, IL-6 may be the best studied and most valuable as a prognostic indicator^9^^,^ 10. Besides that, IL-6 has been detected as early marker for postoperative sepsis^11^.

The knowledge about the postoperative inflammatory response and its repercussions on ventilatory function and oxygenation is of utmost importance so that we can avoid as much as possible major disturbances caused by a more intense local or remote inflammation, which can bring consequences of short to long term.

Perioperative detection of increased plasma concentrations of inflammatory cytokines in lung surgery may be used in addition to other clinical predictors to identify patients at risk for postoperative complications^12^.

Different networks existing between pro-inflammatory and anti-inflammatory mediators lead to adequate responses to infection. The pattern of cytokines released into the microenvironment dictates the direction of the immune reaction for the type of response^13^.

Biomarkers, including cytokines, can aid in the diagnosis, prognosis, and prediction of response to treatment in a wide range of disease settings. The identification of biomarkers can help diagnose the disease, assist in the prognosis and/or predict the outcome of the treatment^14^.

Ghanim et al.^15^ found that elevated inflammatory markers reflected a poor prognosis in patients undergoing lung metastasectomy for colorectal cancer, and a pro-inflammatory state, reflected by high fibrinogen and high C-reactive protein, is a negative factor in the prognosis in patients undergoing curative lung metastasectomy for colorectal cancer. The present study shows significant short-term elevations in IL-6 and IL-10 with a tendency to return to baseline levels in the period studied.

Regarding the analysis of ILs in the present study, it was possible to analyze through the enzyme-linked immunosorbent assay (ELISA) kits only IL-8, and the analyzed ILs were not to be able to read by the ELISA kits in most of the times. Multiplex (micro-assays) uses small samples and a large scope of detection of biomarkers, presenting greater sensitivity when compared to ELISA. The ELISA immunoassay is the traditional method of accurately quantifying cytokine levels. However, it is limited by the fact that only one cytokine can be measured at a time. In contrast to ELISA, micro-assays can evaluate several cytokines simultaneously. They require smaller sample volumes than ELISA, they are more sensitive and they have a greater dynamic range. These factors make micro-assays a cheaper and potentially favorable alternative for the large-scale detection of known proteins^16^.

In this study, we were able to observe the profile of the main ILs analyzed in patients in the post-operative period of uncomplicated pulmonary resection surgery, which could serve as a parameter for comparisons with patients who have evolved with postoperative complications and thereby establish more effective therapeutic interventions, at different postoperative moments, in order to modulate the intensity of the inflammatory response.

In all patients, we can see that PaO_2_/FiO_2_ ratio was not significantly different, and they did not need vasoactive drugs. There was an increase in the PaO_2_/FiO_2_ ratio in the immediate postoperative period, because the patients were still on mechanical ventilation. In the fourth hour after surgery, they were already breathing spontaneously. Even so, no significant change was observed in relation to the preoperative period, and, even if there were, all measurements were above 300, according to clinically normal limits.

In this study, we were able to observe the repercussion on platelet levels in the inflammatory response. The platelet count showed a significant decrease in 48 hours, which may suggest a worsening of the coagulation system associated with an increase in IL-1β and TNF-α. However, the significant reduction in platelet count in the present study still remained according to normal levels, perhaps because the sample was composed of uncomplicated patients.

The leukocyte count showed a significant increase in relation to the preoperative period in the times: immediate postoperative and 4 hours postoperative. Concomitantly, there was an increase in lactate at 4 hours post-surgery. These values suggest that the inflammatory response may have been greater at these times, which also resulted in an increase in HR of up to 16% compared to baseline, but with values according to normal limits.

In the present study, it was possible to notice that TNF-α and IL-1β showed similar behaviors, although not significant, with an increase in 48 hours after surgery. Perhaps with a larger sample of patients it would be possible to identify data with statistical differences. These changes should draw our attention, especially that the patient begins to show elevation of these cytokines even after 48 hours after surgery, demonstrating that the risk of a more aggressive inflammatory response may come later.

The cytokines IL-6 and IL-8 increased in 4 hours after surgery, although it was not statistically significant in IL-8 in relation to the preoperative period.

Cytokine levels, especially IL-6 and IL-8, were associated with postoperative infections and systemic inflammatory response syndrome, which itself was a predictive factor of the duration of the hospital stay^17^.

Bastin et al.^18^ showed patients undergoing lung resection, and one-lung ventilation exhibited significant postoperative inflammation and oxidative damage. The increase in plasma IL-6 after lung resection and one-lung ventilation is consistent with previous reports.

Fink-Neuboeck et al.^19^ suggest that IL-6 is a reliable predictor of postoperative systemic inflammatory response syndrome, and it is able to detect postoperative system inflammatory response syndrome before the onset of related clinical symptoms. When identifying patients at high risk, it would be beneficial to include IL-6 in conventional postoperative monitoring, particularly after extended surgical resection.

Matesanz et al.^20^ identify that plasma levels of IL-6 increase rapidly after surgery, and its monitoring after thoracic surgery non-cardiac can be beneficial in identifying patients increased risk of postoperative inflammatory complications long before its clinical onset.

In the study by Breunig et al.^21^, IL-6 and IL-1RA increased early in the blood, at the end of surgery.

De la Gala et al.^22^ showed that the significant increase in plasma cytokines proves the existence of a systemic inflammatory response in patients undergoing lung resection surgery.

In contrast, IL-10 showed a significant increase in immediate postoperative. Because it is an anti-inflammatory cytokine, whose main effect is to inhibit the synthesis of other cytokines, it already shows significant elevations during the intraoperative period, already preparing the body and preventing it from having a very aggressive reaction in the production of cytokines.

In the study by Leite et al.^8^, the authors showed that the lungs of rats had inflammatory responses after reexpansion after bronchial occlusion, characterized by edema formation, neutrophil recruitment, and increased pulmonary myeloperoxidase activity, which is accompanied by higher levels of IL-6, IL-1, and/or TNF in bronchoalveolar lavage. In addition, the local lung injury was accompanied by a degree of systemic inflammation detected by increased serum concentration IL-6 and IL-10 levels.

Kaufmann et al.^12^ demonstrated that increased IL-6, IL-8, and IL-10 levels at an early stage after lung surgery might be of essential prognostic value to identify patients at risk for postoperative complications. They identified that the multivariate regression analysis of lung surgery patients with increased IL-8 and IL-6 levels above the third quartile on the first postoperative day revealed a significant probability increase for postoperative complications. Apart from that, patients with postoperative complications showed a significantly increased IL-6 and IL-10 plasma concentration already at the time of wound closure compared to those without complications.

Sugasawa et al.^23^ used a bronchoscopic microsampling method to obtain epithelial lining fluid from each lung and compared the inflammatory reactions in the dependent lung and the nondependent lung during thoracic surgery. They showed that level (epithelial lining fluid) of IL-6 was significantly higher in the dependent lung than in the nondependent lung at the end of surgery (*p* = 0.019).

Better knowledge of the inflammatory response will allow customization and personalization of the treatment. A distinct pattern of cytokine levels measured at the beginning of sepsis predicts the course of the disease. The crucial first hours in sepsis determine the course of the disease. When associated with organ failure and shock, this initial phase is marked by an intense inflammatory response and system disorders: endocrine, coagulation, renal, and cardiopulmonary.

Our focus was to evaluate the acute response, but the inflammatory response as well can influence in the medium and long term the survival and the rate of tumor recurrence of patients undergoing surgical treatment or not. Better knowledge and the inflammatory profile can interfere with the oncological evolution of these patients, reducing inflammation and, consequently, maintaining the state of the individual’s innate immunity.

## Conclusion

Patients undergoing uncomplicated lung resection surgery showed an increase in pro-inflammatory IL-6 after 4 hours of surgical intervention and an increase in anti-inflammatory IL-10 in the immediate postoperative period. They also showed an increase in the number of leukocytes, a decrease in the number of platelets, an increase in blood lactate and HR in up to 16% of baseline. There were no changes in the perfusion/oxygenation ratio, but there was a drop in pH due to the increase in carbon dioxide in the first 4 hours of surgery.

The study may contribute to the search for more specific and adequate alternatives for controlling and modulate the inflammatory response in perioperative period. In this way, the intervention would be specific to that cytokine that causes the greatest harm to the patient, as well as to the moment of the intervention.

## Data Availability

The data will be available upon request.
